# Herbicidal Activities of Some Allelochemicals and Their Synergistic Behaviors toward *Amaranthus tricolor* L.

**DOI:** 10.3390/molecules22111841

**Published:** 2017-10-27

**Authors:** Nawasit Chotsaeng, Chamroon Laosinwattana, Patchanee Charoenying

**Affiliations:** 1 Department of Chemistry, Faculty of Science, King Mongkut’s Institute of Technology Ladkrabang, Bangkok 10520, Thailand; patchaneecha@yahoo.com; 2 Department of Plant Production Technology, Faculty of Agricultural Technology, King Mongkut’s Institute of Technology Ladkrabang, Bangkok 10520, Thailand; klchamro@kmitl.ac.th

**Keywords:** allelopathy, herbicidal, xanthoxyline, *R*-(+)-limonene, vanillin, linoleic acid, synergistic, binary mixture, Chinese amaranth

## Abstract

Seven allelochemicals, namely *R*-(+)-limonene (**A**), vanillin (**B**), xanthoxyline (**C**), vanillic acid (**D**), linoleic acid (**E**), methyl linoleate (**F**), and (±)-odorine (**G**), were investigated for their herbicidal activities on Chinese amaranth (*Amaranthus tricolor* L.). At 400 μM, xanthoxyline (**C**) showed the greatest inhibitory activity on seed germination and seedling growth of the tested plant. Both vanillic acid (**D**) and (±)-odorine (**G**) inhibited shoot growth, however, apart from xanthoxyline (**C**), only vanillic acid (**D**) could inhibit root growth. Interestingly, *R*-(+)-limonene (**A**) lightly promoted root length. Other substances had no allelopathic effect on seed germination and seedling growth of the tested plant. To better understand and optimize the inhibitory effects of these natural herbicides, 21 samples of binary mixtures of these seven compounds were tested at 400 μM using 0.25% (*v*/*v*) Tween^®^ 80 as a control treatment. The results showed that binary mixtures of *R*-(+)-limonene:xanthoxyline (**A**:**C**), vanillin:xanthoxyline (**B**:**C**), and xanthoxyline:linoleic acid (**C**:**E**) exhibited strong allelopathic activities on germination and seedling growth of the tested plant, and the level of inhibition was close to the effect of xanthoxyline (**C**) at 400 µM and was better than the effect of xanthoxyline (**C**) at 200 µM. The inhibition was hypothesized to be from a synergistic interaction of each pair of alleochemicals. Mole ratios of each pair of allelochemicals ((**A**:**C**), (**B**:**C**), and (**C**:**E**)) were then evaluated, and the best ratios of the binary mixtures **A**:**C**, **B**:**C** and **C**:**E** were found to be 2:8, 2:8, and 4:6 respectively. These binary mixtures significantly inhibited germination and shoot and root growth of Chinese amaranth at low concentrations. The results reported here highlight a synergistic behavior of some allelochemicals which could be applied in the development of potential herbicides.

## 1. Introduction

Today, undeniably, agricultural crops are very important to humans. However, to achieve high yields of crops, farmers should have a good knowledge of pest prevention and control, especially weed control. Weeds are simply defined as plants growing in an undesired location, competing with crops for resources, lowering crop yields, and contaminating the crop with their seeds [1]. In general, there are numerous methods available to control weeds, and chemical (or herbicide) control is one of the widely used methods [2]. For decades, synthetic herbicides have been used in agriculture as weed killers because they are easy to find, cheap, and display a good weed control. Unfortunately, since the first implementation of these synthetic compounds in crop protection, weeds have incessantly developed resistance against the applied chemicals [3]. Moreover, synthetic herbicides are often toxic and cause environmental concerns [4,5,6,7]. These issues have become a major challenge for many agricultural producers. As such, new strategies are required to cope with weed problems [3,7,8].

**Figure 1:**
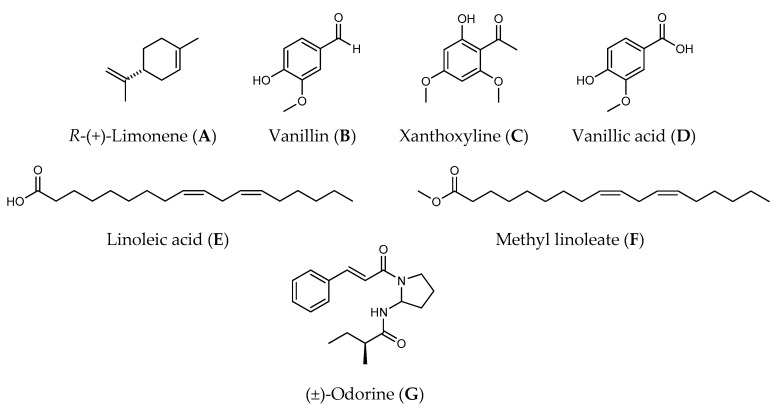
Allelochemicals used in this study.

Presently, the term “allelopathy” is widely applied to control weed plants. It is simply defined as the direct or indirect detrimental or beneficial effects of one plant (including microorganisms) on the germination, growth, or development of other plants through the production of chemicals (allelochemicals) that are released into the environment [9]. The use of allelochemicals as natural herbicides is extensively studied because it is believed that natural products are easy to use, quite safe, environmentally friendly, and rapidly degraded [3,7,8]. There are a number of allelochemicals that have been explored from natural resources since the term allelopathy was coined [1,8,10]. In our research group, for example, we isolated xanthoxyline (**C**) from *Zanthoxylum limonella* Alston fruit which inhibited the seed germination and seedling growth of Chinese amaranth and Barnyard grass [11]. In addition, in 2012 [12,13] we also investigated herbicidal activities of *Aglaia odorata* L. on *Echinochloa crus-galli* and then successively isolated an active bisamide allelochemical, (±)-odorine (**G**) (Figure 1).

It has long been known that some allelochemicals are produced in small quantities by plants, and most of those plants are uncultivated [14]. Moreover, their isolation, purification, and identification are quite difficult and expensive. Therefore, these natural compounds might be insufficient or unsuitable for production as a commercial herbicide. Fortunately, however, several reports [3,7,8,15,16,17,18,19,20,21,22] have revealed that allelopathic effects of plants or microorganisms are occasionally due to synergism of a combination of substances rather than to single compounds. The additive or synergistic interactions are significant, even at low concentrations of the mixtures. This may result from a high structural diversity of the complex combinations.

In order to understand the allelopathic effect of combined materials and to receive a more reactive natural herbicide, binary mixtures of seven natural molecules (containing a variety of functional groups), namely *R*-(+)-limonene (**A**) [23,24,25], vanillin (**B**) [26,27,28,29,30], xanthoxyline (**C**) [11], vanillinic acid (**D**) [28,31,32,33,34,35], linoleic acid (**E**) [36,37,38,39], methyl linoleate (**F**) [40,41,42,43], and (±)-odorine (**G**) [12,13] (Figure 1), were investigated using Chinese amaranth (*Amaranthus tricolor* L.) as the test plant and Tween^®^ 80 as a surfactant. Chinese amaranth was chosen as a representative dicot plant because it has a high percentage of germination. Moreover, it has a small seed which is quite easy to handle in the lab-scale experiment. Also, several researchers [44,45,46,47] have reported the use of this dicot as a test plant in allelopathic studies. Positive results of this research could be utilized to develop cost-effective natural herbicides in the near future.

## 2. Results and Discussion

### 2.1. Effects of Tween^®^ 80 on Germination and Seedling Growth of Chinese Amaranth

Since most of the tested chemicals in this study are insoluble or have relatively low solubility in distilled water, the inhibitory effects of aqueous solutions of these substances on a test plant may not be sufficiently accurate. Generally, solubility could be one of the major parameters influencing the different herbicidal effects exhibited by allelochemicals. The problem of limited water solubility of most organic compounds results in new methodologies for turning water insoluble chemicals into more soluble chemicals. There are various techniques used for the enhancement of the solubility of poorly soluble compounds, and the use of surfactants is one of the effective procedures. For this research, polysorbate 80 (Tween^®^ 80) surfactant was chosen because it has been widely used in many allelopathic studies [17,48,49,50]. However, the effect of this nonionic surfactant on seed germination and seedling growth of plants should not be neglected as it may inhibit or promote plant growth depending upon its applied concentrations [51].

**Figure 2:**
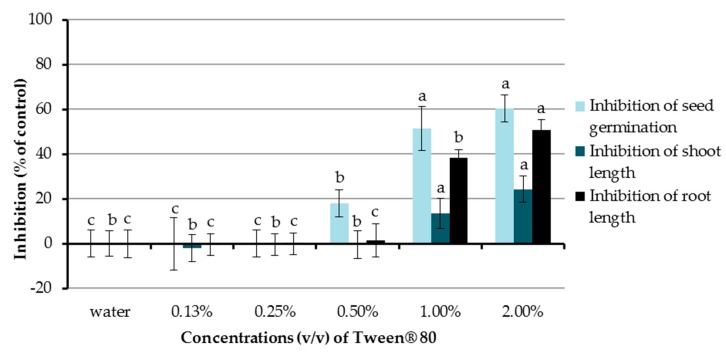
Effects of Tween^®^ 80 (at concentrations of 0.13–2.0% *v*/*v*) on seed germination and seedling growth of Chinese amaranth. Distilled water was used as a control. Means with the same letters in the graph are not significantly different at *p* ≤ 0.05 level.

In the present study, the inhibitory activities of aqueous solutions of Tween^®^ 80 at concentrations of 0.13%, 0.25%, 0.5%, 1.0%, and 2.0% (*v*/*v*) were investigated (Figure 2). Chinese amaranth was used as a test plant and distilled water was used as a control treatment. The results showed that Tween^®^ 80 at 0.5%, 1.0%, and 2.0% (*v*/*v*) significantly inhibited seed germination of the tested plant by 18.15%, 51.51%, and 60.61%, respectively. In terms of seedling growth, Tween^®^ 80 at concentrations of 1.0% and 2.0% (*v*/*v*) could also reduce shoot length (13.73% and 24.42%) and root length (38.48 and 50.79%). Other concentrations of Tween^®^ 80 had no significant effect on seed germination and the growth of seedlings of Chinese amaranth. From the above-mentioned results, the aqueous solution of Tween^®^ 80 at 0.25% (*v*/*v*) exhibited no inhibitory effect on the tested plant, and it was well mixed with all tested compounds; therefore, it was further used as a surfactant and a control treatment in the following sections.

### 2.2. Allelopathic Effects of Seven Allelochemicals and Their Binary Mixtures on Germination and Seedling Growth of Chinese Amaranth

There are seven allelochemicals used in this study and each of them has different chemical structures and functional groups which may pose different biological activities. The herbicidal effects of these natural compounds on certain plants and algae have been well documented, as previously mentioned in the introduction. In this section, we discuss the evaluation of the allelopathic effect of the pure allelochemicals in comparison with their binary mixtures on seed germination and seedling growth of Chinese amaranth in the hope of finding synergism in a binary mixture of these compounds.

**Figure 3:**
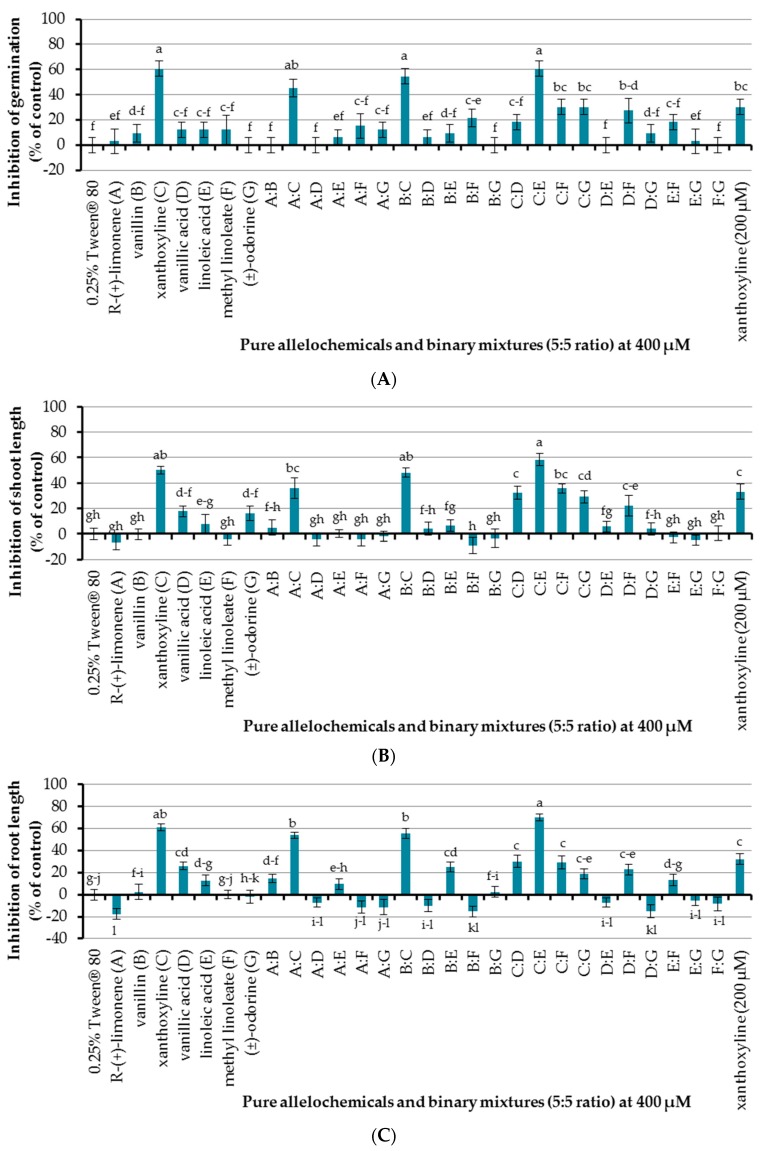
Inhibitory effects of seven allelochemicals and their binary mixtures (at 400 µM) on seed germination (**A**) and shoot (**B**) and root (**C**) growth of Chinese amaranth. A 0.25% aqueous solution of Tween^®^ 80 was used as a control. Means with the same letters in each graph are not significantly different at *p* ≤ 0.05 level.

The effect of seven allelochemicals was preliminarily investigated at a concentration of 400 µM using an aqueous solution of Tween^®^ 80 at 0.25% (*v*/*v*) as a surfactant and control experiment (Figure 3). It was found that only xanthoxyline (**C**) inhibited seed germination of Chinese amaranth, by 60.61%, other pure allelochemicals showed no effect. In terms of seedling growth, xanthoxyline (**C**) and vanillic acid (**D**) inhibited shoot length of Chinese amaranth by 50.09 and 17.76%, respectively, but other compounds had no significant effect on shoot growth. For root length, xanthoxyline (**C**) and vanillic acid (**D**) inhibited root length of Chinese amaranth by 60.68 and 25.95%, respectively. However, other compounds did not inhibit root length. Interestingly *R*-(+)-limonene (**A**) promoted root growth by 17.82% in comparison with control treatment. Overall, looking at all the effects of these natural molecules, xanthoxyline (**C**) exhibited the highest phytotoxicity on Chinese amaranth.

An initial attempt for allelopahic effect of binary mixtures (5:5 ratio) of seven natural compounds at a concentration of 400 µM was performed using 0.25% (*v*/*v*) Tween^®^ 80 as a control treatment. It was shown that binary mixtures **A**:**C**, **B**:**C**, and **C**:**E** inhibited seed germination of Chinese amaranth by 45.45%, 54.55%, and 60.61%, respectively, which is similar to that of xanthoxyline (**C**) at 400 µM. Although xanthoxyline (**C**) at 200 µM also showed inhibition of germination, the result was not significantly different from that of the binary mixture **A**:**C**. For the rest of binary mixtures, only **B**:**F**, **C**:**F**, and **C**:**G** inhibited seed germination of the tested plant, but the effects were slightly lower than that of xanthoxyline (**C**) at 200 µM. Other binary mixtures had no significant effect on seed germination.

In the case of shoot growth, binary mixtures **B**:**C** and **C**:**E** showed high percentages of inhibition by 45.13% and 54.26%, respectively, which was close to the effect of xanthoxyline (**C**) at 400 µM. Binary mixtures **A**:**C**, **C**:**D**, **C**:**F**, **C**:**G**, and **D**:**F** inhibited shoot length by 22.02–36.06% which was close to that of xanthoxyline (**C**) at 200 µM that reduced shoot growth by 33.21%. However, other binary mixtures had no significant effect on shoot length.

Binary mixtures **A**:**C**, **B**:**C**, and **C**:**E** inhibited root length of Chinese amaranth by 53.74%, 55.57%, and 70.12% respectively, which was close to that of xanthoxyline (**C**) at 400 µM. Binary mixtures **B**:**E**, **C**:**D**, **C**:**F**, **C**:**G**, and **D**:**F** inhibited root length by 18.74–30.14% which was similar to xanthoxyline (**C**) at 200 µM that inhibited root growth by 32.24%. However, a binary mixture of **A**:**B** showed a low level of inhibition (14.68%). Other binary mixtures had no significant inhibitory effect on root length but, noticeably, binary mixtures **B**:**F** and **D**:**G** promoted root length of Chinese amaranth by 15.33% and 15.07%, respectively.

From the results mentioned above, only the full dose of xanthoxyline (**C**) alone and a half dose of xanthoxyline (**C**) in binary combinations with *R*-(+)-limonene (**A**), vanillin (**B**), or linoleic acid (**E**) exhibited high inhibitory activity on germination and seedling growth of the tested plant. In comparison with xanthoxyline (**C**) at 200 µM, the allelopatic potentials of these binary mixtures were higher. However, other allelochemicals and other ratios of binary mixtures displayed low or no inhibitory effect on germination and seedling growth of Chinese amaranth. The greater inhibition of binary mixtures may be due to a synergistic behavior of each pair of alleochemicals. Thus, in the next section, ratios of pairs (*R*-(+)-limonene:xanthoxyline (**A**:**C**), vanillin:xanthoxyline (**B**:**C**) and xanthoxyline:linoleic acid (**C**:**E**)) of allelochemicals were investigated to determine the highest inhibitory activity of these three binary mixtures.

### 2.3. Allelopathic Effects of Binary Mixtures (In Different Ratios) of Some Allelochemicals on Germination and Seedling Growth of Chinese Amaranth

#### 2.3.1. Allelopathic Effects of *R*-(+)-limonene (**A**) and Xanthoxyline (**C**) Mixtures on Germination and Seedling Growth of Chinese Amaranth

**Figure 4:**
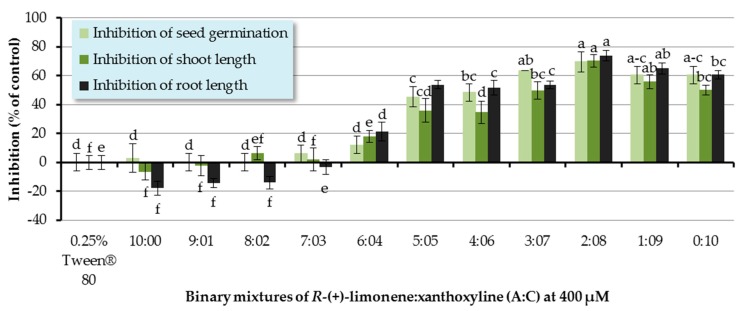
Inhibitory effects of binary mixtures (at 400 µM) of *R*-(+)-limonene (**A**) and xanthoxyline (**C**) on seed germination and shoot and root growth of Chinese amaranth. A 0.25% aqueous solution of Tween^®^ 80 was used as a control. Means with the same letters in the graph are not significantly different at *p* ≤ 0.05 level.

The ratios of a binary mixture of *R*-(+)-limonene:xanthoxyline (**A**:**C**) tested were 9:1–1:9. Each ratio of **A**:**C** had an overall concentration of 400 µM. Pure *R*-(+)-limonene (**A**) and pure xanthoxyline (**C**) at 400 µM were used for comparison, and Tween^®^ 80 at 0.25% (*v*/*v*) was used as a control experiment (Figure 4). It was found that binary mixtures at ratios of 5:5–1:9 inhibited seed germination of Chinese amaranth by 45.45–69.70%. However, this result was not significantly different from that of pure xanthoxyline (**C**, 0:10). For shoot growth, binary mixtures of *R*-(+)-limonene:xanthoxyline (**A**:**C**) at the ratios of 6:4–1:9 showed great inhibitory activity on shoot length. The binary mixture at the ratio of 2:8 significantly inhibited shoot length by 70.34% in comparison with that of pure xanthoxyline (**C**). In term of root growth, binary mixtures of *R*-(+)-limonene:xanthoxyline (**A**:**C**) at ratios of 6:4–1:9 greatly inhibited root length of the tested plant. The mixture at a ratio of 2:8 significantly inhibited root growth by 73.79% compared to pure xanthoxyline (**C**). Interestingly, however, pure *R*-(+)-limonene (**A**) and binary mixtures of **A**:**C** at 9:1 and 8:2 slightly promoted root length.

The above mentioned results demonstrated the synergistic interaction between the nonpolar compound *R*-(+)-limonene (**A**) and the polar compound xanthoxyline (**C**). Similarly, in 2014, Cardoso and co-workers [52] reported the allelopathic effect of essential oils extracted from *Callistemon viminalis* against *Lactuca sativa* L. This herbicidal effect was believed to be due to the presence of the principal constituents 1,8-cineole, α-pinene, and limonene (**A**) or to chemical synergism between the compounds in the essential oil. In terms of compounds with different polarity, the synergistic behavior was previously found in a mixture of a more polar lyoniside and less polar triterpene acids [22]. Besides, it was found here that ratios of the binary mixture (**A**:**C**) were vital to an herbicidal activity, with the ratio of 2:8 (**A**:**C**) exhibiting the best result. This is in agreement with the report by Dilipkumar [53] that synergistic action of aqueous extracts of sunflower leaves and commercial herbicides (pretilachlor and thiobencarb) was combination ratio-dependent. Interestingly, our results showed that binary mixtures of **A**:**C** at 9:1 and 8:2 slightly promoted root length. It frequently appears in literature that phenolic compounds at low concentrations stimulate germination and seedling growth of plants [31,54], but at high concentrations they reduce seed germination sharply.

#### 2.3.2. Allelopathic Effects of Vanillin (**B**) and Xanthoxyline (**C**) Mixtures on Germination and Seedling Growth of Chinese Amaranth

**Figure 5:**
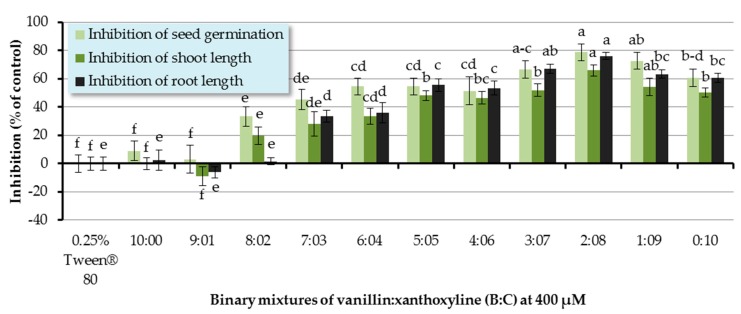
Inhibitory effects of binary mixtures (at 400 µM) of vanillin (**B**) and xanthoxyline (**C**) on seed germination and shoot and root growth of Chinese amaranth. A 0.25% aqueous solution of Tween^®^ 80 was used as a control. Means with the same letters in the graph are not significantly different at *p* ≤ 0.05 level.

Allelopathic effects of binary mixtures of vanillin:xanthoxyline (**B**:**C**) at ratios of 9:1–1:9 were also assessed (Figure 5). It was shown that the binary mixtures at the ratios of 8:2–1:9 significantly inhibited seed germination of Chinese amaranth. The mixture at the ratio of 2:8 had the highest inhibitory effect of germination by 78.79%. In terms of shoot growth, binary mixtures of **B**:**C** at 8:2–1:9 significantly inhibited shoot length. Again, the binary mixtures at the ratio of 2:8 had the highest inhibitory activity on shoot growth by 65.90%. For root growth, binary mixtures at the ratios of 7:3–1:9 showed a strong inhibition on root length. The greatest herbicidal effect was observed in the binary mixture at the ratio of 2:8 which reduced root length by 76.15%. However, other ratios had no significant effect on seed germination and seedling growth of Chinese amaranth.

In this section, there was a synergistic behavior observed between a phenolic aldehyde (vanillin (**B**)) and a phenolic ketone (xanthoxyline (**C**)). This is consistent with the antialgal activities of some phenolic compounds on two strains of *Microcystis aeruginosa* [55]. It was revealed that positions and numbers of hydroxyl groups influenced the effects of phenolic acids. Also, the mixtures of these phenolic allelochemicals showed synergism against toxic *M. aeruginosa*. Moreover, in 2012, Zhang’s group [56] investigated the alleopathic effects of *Typha angustifolia* L. on phytoplankton and noted that phenolic acids existing in the herbaceous plant exerted synergistic inhibitory effects on the growth of the phytoplankton assemblage. Similarly, Chou and coworkers [57] conducted research on the allelopathic effects of (−)-catechin and its microbial transformation substance, protocatechuic acid, against lettuce. They found that protocatechuic acid exhibited higher phytotoxicity than (−)-catechin did, and the effect of (−)-catechin was enhanced by combining it with protocatechuic acid.

#### 2.3.3. Allelopathic Effects of Xanthoxyline (**C**) and Linoleic Acid (**E**) Mixtures on Germination and Seedling Growth of Chinese Amaranth

**Figure 6:**
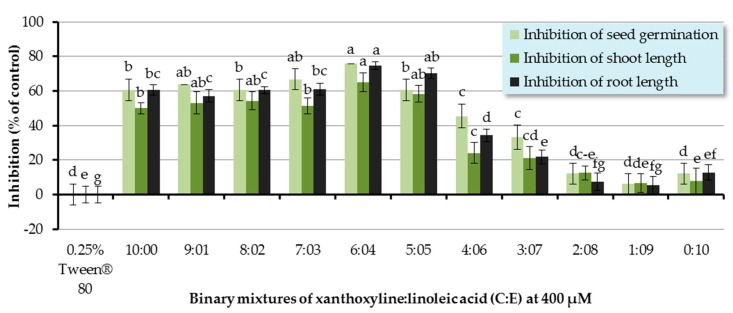
Inhibitory effects of binary mixtures (at 400 µM) of linoleic acid (**E**) and xanthoxyline (**C**) on seed germination and shoot and root growth of Chinese amaranth. A 0.25% aqueous solution of Tween^®^ 80 was used as a control. Means with the same letters in the graph are not significantly different at *p* ≤ 0.05 level.

The inhibitory effect of binary mixtures of xanthoxyline:linoleic acid (**C**:**E**) at the ratios of 9:1–1:9 was also investigated, and the binary mixtures at ratios of 9:1–3:7 significantly inhibited germination and seedling growth of Chinese amaranth (Figure 6). The most active mixture was at the ratio of 4:6 which inhibited germination and shoot and root length of the tested plant by 75.76%, 65.01%, and 74.71%, respectively. However, other ratios had no significant effect.

Since the discovery of synergism among fatty acids by Wallace and Whitehand [58], numerous reports have been published. For example, in 2009, Zhang and coworkers [59] investigated the allelopathic effects of *Chara vulgaris* on the growth and development of toxic *Microcystis aeruginosa* and found that the *C. vulgaris* allelochemicals included three fatty acids, namely linoleic acid (**E**), tetradecanoic acid, and hexadecanoic acid. These three allelochemicals in combination could exert synergistic inhibitory effects on the growth of *M. aeruginosa*. Similarly, our results show the synergism between xanthoxyline (**C**) and linoleic acid (**E**), which is in line with the finding of Zuo and coworkers [60]. They evaluated the inhibitory effects of five allelochemicals (coumarin, *p*-hydroxybenzoic acid, protocatechuic acid, stearic acid, and *p*-aminobenzenesulfonic acid) on the growth of *Chlorella pyrenoidosa*. It was shown that individual allelochemicals had strong algal inhibition effects. Moreover, they also highlighted that the specific proportions of two or three allelochemicals could display synergistic allelopathic interactions.

From the above results, binary mixtures of **A**:**C** (2:8), **B**:**C** (2:8), and **C**:**E** (4:6) showed the highest inhibitory activity on seed germination and seedling growth of Chinese amaranth. The results were significantly different from that of xanthoxyline (**C**) at 400 µM. This confirmed a synergistic behavior of each pair of allelochemicals and revealed the most suitable ratios of binary mixtures. As such, the next section discusses the allelopathic effect of binary mixtures of *R*-(+)-limonene:xanthoxyline (**A**:**C**, 2:8), vanillin:xanthoxyline (**B**:**C**, 2:8), and xanthoxyline:linoleic acid (**C**:**E**, 4:6), which were investigated in order to find the minimum concentration at which that these binary mixtures could inhibit seed germination and seedling growth of Chinese amaranth.

### 2.4. Allelopathic Effects of Best Fractions of Binary Mixtures of Some Allelochemicals on Germination and Seedling Growth of Chinese Amaranth

#### 2.4.1. Allelopathic Effects of a Binary Mixture of *R*-(+)-Limonene:Xanthoxyline (2:8) on Germination and Seedling Growth of Chinese Amaranth

**Figure 7:**
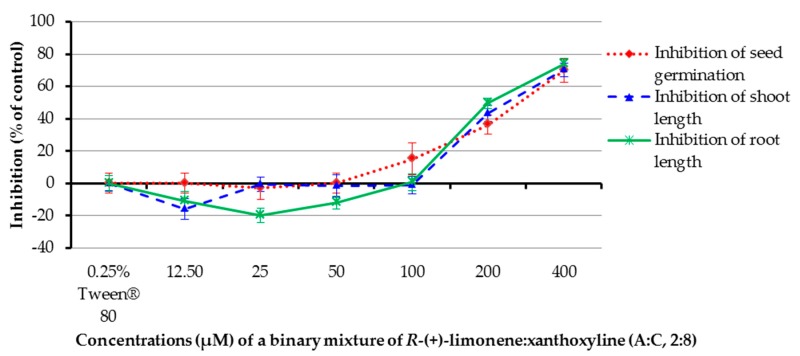
Inhibitory effects of a binary mixture of *R*-(+)-limonene:xanthoxyline (2:8) on seed germination and shoot and root growth of Chinese amaranth. A 0.25% aqueous solution of Tween^®^ 80 was used as a control.

Binary mixtures of *R*-(+)-limonene:xanthoxyline (**A**:**C**, 2:8) at concentrations of 12.5–400 µM were tested, and a 0.25% (*v*/*v*) aqueous solution of Tween^®^ 80 was a control experiment. It was revealed that only concentrations of 200 and 400 µM showed a strong inhibition on seed germination of Chinese amaranth (Figure 7). The solution at concentrations of 200 and 400 µM could inhibit shoot growth. Interestingly, shoot length of Chinese amaranth was slightly promoted by the mixture at 12.5 µM. Again, in the case of root growth, the binary mixture at concentrations of 200 and 400 µM inhibited root length. However, at concentrations of 12.5–50 µM root length of the tested plant was promoted by 11.01–20.05%. The result in this section highlights that the inhibitory effect of this binary mixture is directly proportional to the increasing concentrations.

#### 2.4.2. Allelopathic Effects of a Binary Mixture of Vanillin:Xanthoxyline (2:8) on Germination and Seedling Growth of Chinese Amaranth

**Figure 8:**
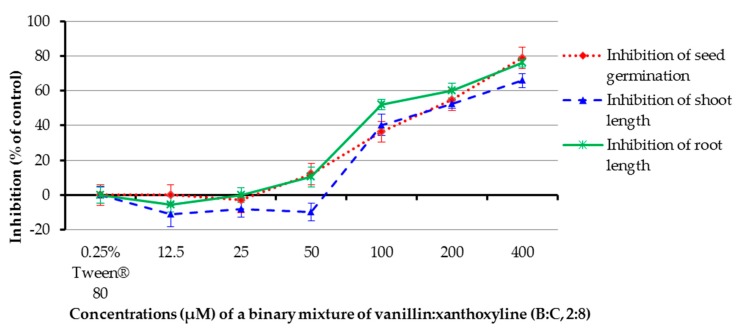
Inhibitory effects of a binary mixture of vanillin:xanthoxyline (2:8) on seed germination and shoot and root growth of Chinese amaranth. A 0.25% aqueous solution of Tween^®^ 80 was used as a control.

The allelopathic effect of a binary mixture of vanillin:xanthoxyline (**B**:**C**, 2:8) at 12.5–400 µM was studied (Figure 8). It was found that the mixture at concentrations of 100, 200, and 400 µM inhibited germination of the tested plant by 36.36%, 54.56%, and 78.79%, respectively, and inhibited shoot length by 40.32%, 52.40%, and 65.90%, respectively. In contrast, at concentrations of 12.5–50 µM the mixture had low or no effect on germination and shoot growth. The mixture at 50–400 µM inhibited root growth by 10.35–76.15%, but other concentrations had no herbicidal effect. Again, the result here revealed that the percentage of inhibition within seven days increased with increasing concentrations of the mixture.

#### 2.4.3. Allelopathic Effects of a Binary Mixture of Xanthoxyline:Linoleic Aicd (4:6) on Germination and Seedling Growth of Chinese Amaranth

**Figure 9:**
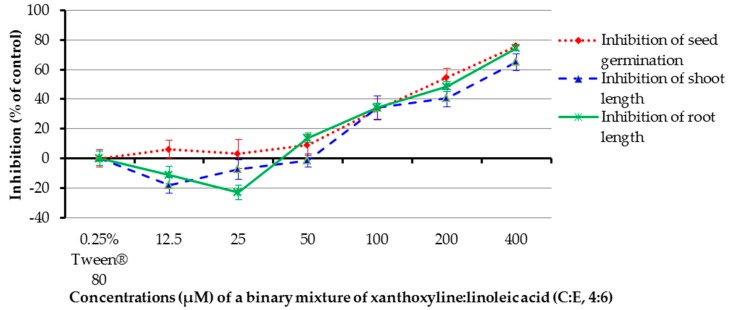
Inhibitory effects of a binary mixture of xanthoxyline:linoleic aicd (4:6) on seed germination and shoot and root growth of Chinese amaranth. A 0.25% aqueous solution of Tween^®^ 80 was used as a control.

For the effect of a binary mixture of xanthoxyline:linoleic acid (**C**:**E**, 4:6), it was shown that at concentrations of 100–400 µM, the binary mixture inhibited germination of the tested plant by 33.33–75.76% and inhibited shoot length by 34.10–65.01% (Figure 9). In contrast, at a concentration of 12.5 µM the mixture stimulated seed germination by 18.12%. However, other ratios had a low effect on seed germination and shoot growth of Chinese amaranth. The mixture at 50–400 µM inhibited root growth by 13.89–74.71%. Surprisingly, at the concentrations of 12.5 and 25 µM the mixture promoted root length by 11.12% and 22.94%, respectively. This again pointed out that the allelopathic effect of the binary mixture is concentration–dependent.

In this section, the minimum concentrations of binary mixtures between xanthoxyline (**C**) and the other three natural products (**A**, **B**, and **E**) were revealed. The seed germination and seedling growth of Chinese amaranth were still inhibited at very low concentrations of tested chemicals. The promising results from laboratory-scale investigations could be further applied to the development of natural herbicides at an industrial level in the future.

## 3. Experimental

### 3.1. Chemicals and Instrument

*R*-(+)-limonene (**A**) and Tween^®^ 80 were purchased from Sigma-Aldrich (Singapore). Vanillin (**B**), vanilinic acid (**D**), linoleic acid (**E**), and methyl linoleate (**F**) were purchased from Fluka (Buchs, Switzerland). All five commercially available allelochemicals were reagent grade and used without further purification. All solvents were purified before use, according to standard procedures. All NMR spectra were recorded on a Bruker Avance III HD (500 MHz) (Bruker BioSpin GmbH, Rheinstetten, Germany) at Scientific and Technological Research Equipment Center, Chulalongkorn University (Bangkok, Thailand). Chemical shifts (δ) are quoted in parts per million (ppm) downfield of tetramethylsilane, using residual protonated chloroform (CDCl_3_) as internal standard (7.27 ppm for ^1^H NMR and 77.00 ppm for ^13^C NMR).

### 3.2. Isolation of Xanthoxyline *(****C****)*

Pure xanthoxyline (**C**) was isolated from dried fruits of *Zanthoxylum limonella* as we previously described [11]. One kilogram of the dried fruits of *Z. limonella* was extracted with hexane and ethyl acetate. After filtration and evaporation to get rid of the solvents, crude hexane and crude ethyl acetate were obtained. The crude ethyl acetate was then subjected to silica gel column chromatography using 2% ethyl acetate in hexane as an eluent to give a fraction containing a nearly pure xanthoxyline (**C**). This fraction was then recrystallized in hexane/ethyl acetate to afford pure xanthoxyline (**C**) as a white crystal after filtration. ^1^H NMR (500 MHz, CDCl_3_) δ 14.02 (1H, s, OH), 6.04 (1H, d, *J* = 2.4 Hz, ArH), 5.91 (1H, d, *J* = 2.4 Hz, ArH), 3.84 (3H, s, OCH_3_), 3.81 (3H, s, OCH_3_), 2.60 (3H, s, CH_3_); ^13^C NMR (125.5 MHz, CDCl_3_) δ 203.1 (C=O), 167.5 (**C**), 166.0 (**C**), 162.9 (**C**), 105.9 (**C**), 93.4 (CH), 90.6 (CH), 55.5 (2 × CH_3_), 32.8 (CH_3_). The NMR data were in agreement with the literature [11,61].

### 3.3. Isolation of (±)-Odorine *(****G****)*

Pure (±)-odorine (**G**) was isolated from dried leaves of *Aglaia odorata* L. as we previously described [13]. One kilogram of the air-dried leaves of *A. odorata* L. was ground and then sequentially extracted with hexane and ethyl acetate. After filtration and evaporation to get rid of the solvents, crude hexane and crude ethyl acetate were obtained. The crude ethyl acetate was then subjected to silica gel column chromatography using gradient eluents (ethyl acetate-hexane) as a mobile phase to give a fraction containing a nearly pure (±)-odorine (**G**) at 30% ethyl acetate in hexane as an eluent. This fraction was then recrystallized in hexane/ethyl acetate to afford pure (±)-odorine (**G**) as a white solid after filtration. ^1^H NMR (500 MHz, CDCl_3_) δ 7.66 (1H, d, *J* = 15.4 Hz, CH=CH), 7.50–7.56 (2H, m, ArH), 7.32–7.39 (3H, m, ArH), 6.93 (1H, d, *J* = 15.4 Hz, CH=CH), 6.08–6.15 (1H, m, CH), 3.57–3.68 (1H, m, CH_2_), 3.37–3.45 (1H, m, CH_2_), 2.12–2.24 (2H, m, CH_2_), 1.84–2.00 (2H, m, CH_2_), 1.59–1.71 (1H, m, CH), 1.34–1.44 (2H, m, CH_2_), 1.15 (3H, d, *J* = 6.9 Hz, CH_3_), 0.79 (3H, t, *J* = 7.4 Hz, CH_3_); ^13^C NMR (125.5 MHz, CDCl_3_) δ 175.7 (C=O), 165.8 (C=O), 142.9 (CH), 134.8 (**C**), 129.8 (CH), 128.8 (2 × CH), 128.2 (2 × CH), 118.0 (CH), 62.8 (CH), 46.1 (CH_2_), 43.0 (CH), 34.5 (CH_2_), 26.9 (CH_2_), 21.5 (CH_2_), 17.5 (CH_3_), 11.8 (CH_3_). The NMR data were in agreement with the literature [13].

### 3.4. Preparation of Aqueous Solutions of Tween^®^ 80

Two milliliters of Tween^®^ 80 and 40 mL of distilled water were added to a 100 mL–beaker. The mixture was stirred at room temperature for 10 min until it well mixed or became a clear solution. The solution was then transferred to a 100 mL–volumetric flask and distilled water was added to the flask to adjust the volume to 100 mL. The solution was mixed thoroughly by stoppering the flask securely and inverting it several times for 2 min to afford an aqueous stock solution of 2% (*v*/*v*) Tween^®^ 80. A 1% (*v*/*v*) aqueous solution of Tween^®^ 80 was prepared by pipetting 50 mL of the stock solution into a 100 mL volumetric flask, diluting it with distilled water until the solution reached the mark, and inverting the mixture gently. Other concentrations (0.5%, 0.25%, and 0.13% (*v*/*v*)) were prepared in a similar dilution procedure to afford the aqueous solutions of Tween^®^ 80.

### 3.5. Preparation of Aqueous Solutions of Seven Allelochemicals and Their Binary Mixtures (5:5 Mole Ratio)

Forty micromoles of a pure allelochemical and 0.25 mL of Tween^®^ 80 were mixed in a 100 mL–beaker until it became a clear solution. Forty milliliters of distilled water was then added to the beaker and the mixture was stirred at room temperature for 10 min until it was thoroughly mixed. The solution was then transferred to a 100 mL–volumetric flask and distilled water was added to the flask to adjust the volume to 100 mL. This solution was a 400 μM aqueous solution of a pure allelochemical which contained 0.25% (*v*/*v*) of Tween^®^ 80.

A binary mixture (5:5 mole ratio) of two allelochemicals was prepared by pipetting 50 mL of a 400 μM aqueous solution of the first allelochemical to a 100 mL–volumetric flask and then adjusting the volume of the binary mixture with a 400 μM aqueous solution of the second allelochemical (the final solution contained 0.25% (*v*/*v*) of Tween^®^ 80 and 200 μM of each allelochemical). The binary mixtures at mole ratios of 9:1, 8:2, 7:3, 6:4, 4:6, 3:7, 2:8, and 1:9 were prepared by using the same procedure.

### 3.6. Preparation of Aqueous Solutions of R-(+)-Limonene:Xanthoxyline (**A**:**C**, 2:8), Vanillin:Xanthoxyline (**B**:**C**, 2:8), and Xanthoxyline:Linoleic Acid (**C**:**E**, 4:6) at 400, 200, 100, 50, 25, and 12.5 μM

The stock solution of *R*-(+)-limonene:xanthoxyline (**A**:**C**, 2:8) at 400 μM was prepared as described in Section 3.5. An aqueous solution of *R*-(+)-limonene:xanthoxyline (**A**:**C**, 2:8) at 200 μM was prepared by pipetting 50 mL of the 400 μM stock solution of **A**:**C** (2:8) to a 100 mL–volumetric flask and then adjusting the volume of the binary mixture with a 0.25% (*v*/*v*) aqueous solution of Tween^®^ 80 (the final solution contained 0.25% (*v*/*v*) of Tween^®^ 80 and 200 μM of **A**:**C** (2:8)). For the binary mixtures of **A**:**C** (2:8) at concentrations of 100, 50, 25, and 12.5 μM, vanillin:xanthoxyline (**B**:**C**, 2:8) and xanthoxyline:linoleic acid (**C**:**E**, 4:6) at concentrations of 400, 200, 100, 50, 25, and 12.5 μM were prepared with the same dilution procedure.

### 3.7. Tested Plant

Chinese amaranth (*Amaranthus tricolor* L.) was chosen as the test plant for seed germination and seedling growth bioassay. Commercial Chinese amaranth was purchased from Thai Seed & Agriculture Co. Ltd., Bangkok, Thailand. In germination tests, germination activity of these seeds was randomly checked and was found to be >80%.

### 3.8. Seed Germination and Seedling Growth Bioassay

The 0.5 mL of an aqueous solution of Tween^®^ 80 (or aqueous solutions of pure allelochemicals or binary mixtures of allelochemicals) was added into a small vial (4.5 cm × 2 cm) lined with germination paper. Ten seeds of Chinese amaranth were then placed on the germination paper. The vials were sealed with Parafilm^®^ and maintained at 28–30 °C. Control treatments were treated with distilled water or 0.25% (*v*/*v*) aqueous solution of Tween^®^ 80 (0.5 mL/vial). The treatments were replicated four times. Germination and shoot and root lengths were determined after seven days. The percentages of inhibition of seed germination and seedling growth were calculated from the following equation:

$$\mathrm{Inhibition}\;\left(\%\;\mathrm{of}\;\mathrm{control}\right)=100-\frac{\left(\mathrm{pure}\;\mathrm{compound}\;\mathrm{or}\;\mathrm{binary}\;\mathrm{mixture}\right)}{\left(\mathrm{control}\right)}\times 100$$

### 3.9. Statistical Analysis

The control treatments were conducted under the same conditions, in the absence of compounds. A completely randomized design (CRD) with four replications for the variables was employed. Analysis of variance was calculated for all data and comparisons between treatments were made at probability level *p* ≤ 0.05 using Tukey’s studentized range test.

## 4. Conclusions

In the present study, herbicidal activities of seven allelochemicals and their binary mixtures were investigated on a dicotyledon plant, Chinese amaranth. Individually, xanthoxyline (**C**) exhibited the strongest allelopathic effects on seed germination and seedling growth of the test plant. For the binary mixtures, *R*-(+)-limonene:xanthoxyline (**A**:**C**), vanillin:xanthoxyline (**B**:**C**), and xanthoxyline:linoleic acid (**C**:**E**) mixtures at the mole ratios of 2:8, 2:8, and 4:6, respectively, showed the greatest inhibitory activities on the plant germination and growth. The results demonstrated well the synergistic suppressive effect between xanthoxyline (**C**) and the other three natural compounds (alkene (**A**), phenolic aldehyde (**B**), and unsaturated fatty acid (**E**)) in an aqueous solution of surfactant Tween^®^ 80. The inhibitory effect of binary mixtures is dependent on applied concentrations. This finding could be potentially exploited for the development of natural and environmental friendly herbicides in agricultural practices.
